# Simulated phacoemulsification training using the OrbiTau artificial eye: Experience at a single institute

**DOI:** 10.5935/0004-2749.2024-0084

**Published:** 2025-02-11

**Authors:** Tauanni Cândido, Roberto Pineda, Silvana Rossi, Jéssica Véras Moura Lanza, Newton Kara-Júnior

**Affiliations:** 1 Department of Ophthalmology, Faculdade de Medicina, Universidade de São Paulo, São Paulo, SP, Brazil; 2 Thomas Y and Clara W Butler Chair in Ophthalmology, Massachusetts Eye and Ear Infirmary; 3 Department of Ophthalmology, Harvard Medical, Boston, EUA; 4 Hospital Evangélico de Belo Horizonte, Belo Horizonte, MG, Brazil

**Keywords:** Cataract extraction/education, Simulation training/methods, Ophthalmology/education, Phacoemulsification/education, Ophthalmologists/education, Surgeons/education, High fidelity simulation training

## Abstract

**Purpose:**

The OrbiTau surgical simulator is a synthetic eye model developed to enhance cataract surgical training. Herein, we aimed to describe the perspectives of Harvard’s Ophthalmology faculty and residents regarding the effectiveness of OrbiTau.

**Methods:**

A cross-sectional study was conducted in which 11 surgeons from the Massachusetts Eye and Ear Infirmary, with prior experience utilizing simulated phacoemulsification platforms, conducted cataract surgery with the OrbiTau. Subsequently, they completed a satisfaction questionnaire using the Likert scale.

**Results:**

Regarding the various OrbiTau components, 90.90% of the participants reported that the OrbiTau lens capsule was comparable to that of the human lens during capsulotomy. Furthermore, 72.72% of the participants found that the OrbiTau lens consistency was analogous to that of the human lens nucleus. Approximately 63.63% of the participants reported that the model’s posterior lens capsule resembled the native posterior capsule, and 72.72% of the participants noted that the model’s red reflex was similar to that of the dilated human pupil. Most participants believed that the OrbiTau was easier to use and more realistic than other commercially available simulators.

**Conclusion:**

Our single-institution survey of the Orbitau demonstrated that this model realistically replicates ocular structures and may be a viable option for cataract surgery training.

## INTRODUCTION

The use of synthetic tissue eye models has recently gained widespread acceptance in ophthalmic surgical education because of their replicability, potential for reuse, and cost-saving possibilities^(1)^. The OrbiTau surgical simulator is a synthetic eye model designed to train ophthalmologists in phacoemulsification for cataracts. It is manufactured in Brazil and low in cost in comparison with other models such as Bioniko, SimulEye, Phake-I, Kitaro and Eyesi.

In this study, we aimed to elucidate the perspectives of faculty and residents of the University’s ophthalmology department with experience in performing simulated surgeries regarding the effectiveness of the OrbiTau artificial eye model in simulating phacoemulsification.

## METHODS

**Figure 1:**
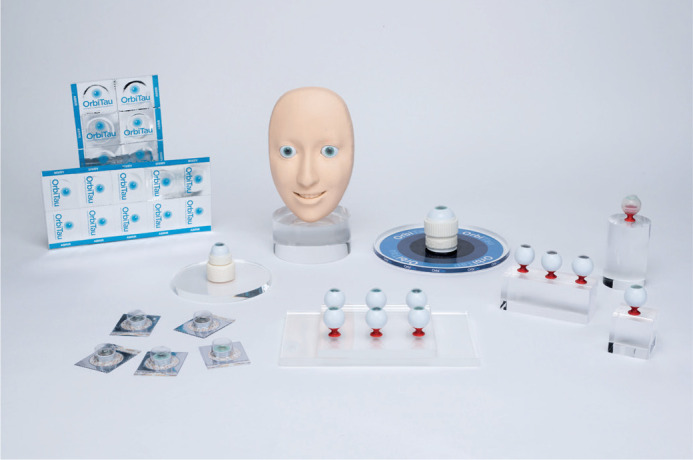
The surgical eye model OrbiTau.

OrbiTau is an artificial eye model that can be used during surgical training to simulate the steps of cataract surgery. This synthetic eye model has a round, transparent upper structure with a convex anterior surface and a concave posterior surface. The dimensions, thickness, elasticity, mechanical resistance, texture, and malleability of the model are similar to those of the human cornea. The model’s anterior lens capsule is slightly elastic with low mechanical resistance and minimal resistance to rupture. The posterior capsule has similar characteristics and is slightly thinner than the anterior capsule. The iris is represented by a highly malleable laminar structure (Figure 1). The OrbiTau eye model allows for training in the steps of cataract surgery as well as the possible surgical complications such as rupture of the posterior capsule, anterior vitrectomy, and secondary implantation of an intraocular lens.

The study was conducted at the Massachusetts Eye and Ear Infirmary, Harvard Medical School. A wet lab was set up on a single day in July 2023, wherein 11 cataract surgeons who have used various commercially available simulators (e.g., Bioniko, SimulEye, Philips Eye, Kitaro, Phake-I and Eyesi) performed phacoemulsification using the OrbiTau simulator. The inclusion criterion was surgeons who have completed >15 h of surgical training using simulators or animal eyes.

To evaluate the effectiveness of the OrbiTau, a 5-point Likert scale^(2)^ was used, which consisted of the following response options: 1, completely disagree or very dissatisfied; 2, disagree or dissatisfied; 3, neutral, 4, agree/satisfied; and 5, completely agree/very satisfied. The participants were allowed 4 h to perform phacoemulsification using the Orbitau simulator.

In this study, the CENTURION Vision System (Alcon, Fort Worth, TX, USA) was used to perform phacoemulsification. After the completion of the surgeries, each participant was asked to fill out a questionnaire regarding the OrbiTau’s usability, structure, similarity to the human eye, and differences from other simulators that they have used. To ensure participants were comfortably in expressing their opinions on the model, no personal information was collected in the questionnaire.

### Ethical considerations

The study was approved by the institution’s Ethics Committee (CEP number: 5.959.616; date: March 23, 2023).

## RESULTS

Approximately 90.90% of the participants reported that the OrbiTau lens capsule was similar to that of a human lens during capsulotomy. Furthermore, 72.72% of the participants found that the consistency of the OrbiTau lens was analogous to that of the human lens nucleus. Approximately 63.63% of the participants reported that the posterior lens capsule resembled the native posterior capsule, and 72.72% of the participants found that the model’s red reflex was similar to that of a dilated human pupil. Most participants agreed that the OrbiTau was easier to use and more realistic than other commercially available surgical eye simulators. The surgeons’ perspectives regarding the degree of similarity between the OrbiTau components and the human eye are presented in table 1. Most participating surgeons indicated that the OrbiTau eye model was highly similar to the human eye (Table 1).

**Table 1:** Perception of the similarity of the OrbiTau structures with the human eye on the basis of previous surgical experience with human eyes

	Totally disagree	Disagree	Neutral	Agree	Totally agree	I can’t evaluate
Cornea	____	9.09%	27.27%	36.36%	18.18%	9.09%
Iris	____	18.18%	36.36%	9.09%	18.18%	18.18%
Anterior lens capsule	____	_____	9.09%	72.72%	18.18%	_____
Lens nucleus	____	_____	27.27%	45.45%	27.27%	_____
Posterior lens capsule	____	9.09%	18.18%	54.54%	9.09%	9.09%
Retinal red reflex	____	_____	18.18%	36.36%	36.36%	9.09%

The reproducibility of the surgical steps using the OrbiTau surgical simulator were evaluated. The incision, capsulorhexis, and phacoemulsification maneuvers stood out for their reproducibility, with satisfaction rates of 90.9%, 90.9%, and 72.72%, respectively. These results demonstrate the effectiveness of the simulator in replicating these surgical steps. Thus, OrbiTau provides a realistic and high-quality experience, especially in the challenging phases of the procedure such as capsulorhexis, fracture, and nucleus capture (Table 2).

**Table 2:** Degree of satisfaction with the precision of carrying out different surgical steps in the OrbiTau on the basis of previous experience with human eyes

Surgical steps	Very unsatisfied	Unsatisfied	Neutral	Satisfied	Very satisfied	I can’t evaluate
Incision	____	____	___	45.45%	45.45%	9.09%
Capsulorhexis	____	____	9.09%	27.27%	63.63%	____
Hydrodissection	____	9.09%	18.18%	27.27%	27.27%	9.09%
Phacoemulsification (Fracture and conquest)	____	____	27.27%	36.36%	36.36%	___

Table 3 highlights the results obtained from the evaluation of the OrbiTau surgical simulator by the study participants. The completed questionnaires revealed a positive feedback and favorable responses regarding the model’s ease of use, representation of ocular structures, and ability to provide comprehensive training (Table 3).

**Table 3:** Overall rating of the OrbiTau

	Totally disagree	Disagree	Neutral	Agree	Totally agree
I liked the OrbiTau surgical simulator	____	____	____	18.18%	82.82%
It is easy to use	____	____	____	9.09%	90.9%
Reproduces the surgical steps well	____	____	____	45.45%	54.54%
The tactile properties are adequate	____	____	9.09%	18.18%	72.72%
It is similar to the form of manipulation in human eyes	___	____	9.09%	27.27%	64.64%

Table 4 shows the advantages of the OrbiTau simulator over the other simulators according to the participants’ previous experiences. Approximately 72.72% of the respondents agreed that OrbiTau was superior to other previously used simulators (e.g., Bioniko, SimulEye, Phake-I, Kitaro, and Eyesi) in terms of usability and representation of ocular structures. Furthermore, they reported that OrbiTau provided a more realistic surgical training experience than the other simulators (Table 4).

**Table 4:** Perceived advantages of the OrbiTau over other simulators (Bioniko, SimulEye, Phake-I, and Kitaro)

	%	I can’t evaluate
Ease of use	72.72%	9.09
Good representation of the ocular structures	72.72%	9.09
Possibility of training for the complete surgery and its complications	72.72%	9.09
Proximity to reality	63.63%	9.09

## DISCUSSION

Cataract is one of the major causes of blindness worldwide, affecting millions of people, especially those aged >60 years^(3^,^4)^. Thus, the surgical training of ophthalmologists performing cataract surgeries is of significant social relevance. Cataract surgery requires dexterity on the part of the ophthalmic surgeon^(3^,^5)^. Therefore, a significant number of ophthalmologists in Latin America abstain from performing cataract surgery, primarily due to concerns of insecurity and inadequate surgical training^(4)^.

Several simulators have been evaluated for their effectiveness in surgical training^(6^-^10)^. According to Belyea et al., residents who trained using a simulator exhibited shorter phacoemulsification times, a lower percentage of phaco energy delivered, fewer intraoperative complications, and a shorter learning curve^(10)^. Dean et al.^(11)^ also highlighted the advantages of using simulators. According to Raval et al.^(12)^, the SimulEYE and Kitaro capsular excision models are similar to human capsular tissue. Studies have also found that training becomes more standardized using a simulated artificial eye than using a live surgical environment^(11)^.

Lucas et al. concluded that training with the Eyesi^®^ cataract surgery simulator significantly reduced the total number of intraoperative complications during the first 10 phacoemulsification surgeries performed by ophthalmology residents^(8)^. Other studies have demonstrated that supervised training on simulators is efficient in providing surgeons with confidence during the learning curve^(13)^.

In our study, most of the participating surgeons agreed that the OrbiTau cornea was similar to the human cornea (Table 1), which probably reflects the predominant perception that the incision maneuver was reproducible (Table 2). The same perspective was noted regarding the anterior lens capsule, capsulorhexis maneuver, consistency of the lens nucleus during phacoemulsification, and the nucleus fracture and disassembly steps, including the aspiration/emulsification of the fragments.

For a cataract surgery simulator to be effective, it should adequately replicate the steps of capsulorhexis and emulsification because they are considered the most difficult to learn and have the greatest potential for complications^(13^-^16)^. In our study with the OrbiTau, the incision, capsulorhexis, and fracture and disassembly stages received a satisfaction level of 90.9%, 90.9%, and 72.72%, respectively, for simulated performance. The participants also expressed appreciation for the replication of the posterior lens capsule, red reflex intensity of the simulator’s retinal component, and the hydrodissection and nucleus rotation maneuvers (Table 2). Overall, the OrbiTau was considered favorably by the ophthalmology surgeons at Harvard (Table 3).

Table 4 shows the main factors that differentiate the OrbiTau surgical simulator from other existing simulators, and it is based on the participants’ previous experience with other surgical models. Among the study participants, 73.73% agreed that the OrbiTau simulator was easy to use, represented the ocular structures well, and allowed for comprehensive surgical training.

Although the study is limited by its small sample size, it is the first to be conducted using the OrbiTau simulator and demonstrates its potential utility in surgical training. Another limitation of the study is that comparisons between the OrbiTau simulator and other available models were based on the participants’ subjective perceptions. Future studies could benefit from a one-on-one comparison between the simulators.

In conclusion, using the OrbiTau simulator allows for safe, reproducible, and practical training in the critical steps of phacoemulsification. This highlights the simulator’s potential to meet the need for a safer and more efficient method of surgical training, especially in regions where access to hands-on training is limited.
